# Renal thrombotic microangiopathy in a patient with septic disseminated intravascular coagulation

**DOI:** 10.1186/1471-2369-14-260

**Published:** 2013-11-27

**Authors:** Yusuke Sakamaki, Konosuke Konishi, Koichi Hayashi, Akinori Hashiguchi, Matsuhiko Hayashi, Eiji Kubota, Takao Saruta, Hiroshi Itoh

**Affiliations:** 1 Department of Internal Medicine, Shizuoka Red Cross Hospital, 8-2 Otemachi, Aoi-Ku, Shizuoka-City, Shizuoka 420-0853, Japan; 2 Division of Endocrinology, Metabolism and Nephrology, Department of Internal Medicine, Keio University, 35 Shinanomachi, Shinjuku-ku, Tokyo 160-8582, Japan; 3 Department of Pathology, Keio University, 35 Shinanomachi, Shinjuku-ku, Tokyo 160-8582, Japan; 4 Center for Apheresis and Dialysis, Keio University, 35 Shinanomachi, Shinjuku-ku, Tokyo 160-8582, Japan

**Keywords:** Thrombotic microangiopathy, Cortical necrosis, DIC, Sepsis, Renal biopsy, TTP, HUS, Plasma exchange, ADAMTS-13

## Abstract

**Background:**

The mechanism for the development of thrombotic microangiopathy (TMA) during sepsis has only been partially elucidated. TMA is recognized as a disease caused by various factors, and may be involved in the emergence of organ damage in severe sepsis. Here we report a case of TMA that followed disseminated intravascular coagulation (DIC) due to severe infection in a patient with a reduced ADAMTS-13 activity level.

**Case presentation:**

An 86-year-old Japanese woman was admitted to our hospital because of low back pain and fever. A careful evaluation led to a diagnosis of acute obstructive pyelonephritis due to a ureteral stone. *Proteus mirabilis* was isolated from both blood and urine cultures. The patient developed systemic inflammatory response syndrome and DIC, and was treated with antibiotics and daily continuous hemodiafiltration. Although infection and the coagulation abnormalities due to DIC were successfully controlled, renal failure persisted and her consciousness level deteriorated progressively in association with severe thrombocytopenia and microangiopathic hemolytic anemia. We therefore suspected the presence of TMA and started plasma exchange, which resulted in an impressive improvement in consciousness as well as the laboratory abnormalities. The ADAMTS-13 activity was 44% and the patient tested negative for the ADAMTS-13 inhibitor prior to the initiation of plasma exchange. A renal biopsy was performed to determine the etiology of acute renal injury, which revealed findings that were interpreted to be compatible with the sequelae of TMA. The follow-up studies performed after the successful treatment of TMA showed that her plasma ADAMTS-13 activity level remained persistently low. It is surmised that septic DIC occurring in the presence of preexisting reduced ADAMTS-13 activity have led to the development of secondary TMA in the present case.

**Conclusion:**

The present case suggests that TMA can be superimposed on sepsis-induced DIC, and plasma exchange is expected to be beneficial in such situations. Clinicians should consider the possibility of secondary TMA that follows sepsis-induced DIC in certain indicative clinical settings.

## Background

Severe infectious disease and systemic inflammatory syndrome (SIRS) often cause disseminated intravascular coagulation (DIC). Thrombotic microvascular occlusion occurring during DIC is known to cause acute kidney injury. Thrombotic thrombocytopenic purpura (TTP) and hemolytic uremic syndrome (HUS) are also known to cause thrombotic microvascular occlusion of various organs including the kidney [1]. TTP and HUS are the primary prototypes of a group of disorders characterized by thrombocytopenia due to increased platelet aggregation and microangiopathic hemolytic anemia collectively called thrombotic microangiopathy (TMA). DIC and TMA are recognized as distinct disease states from the viewpoint of the mechanism responsible for vascular occlusion; DIC is characterized by activation of coagulation pathways and excessive thrombin generation with subsequent consumption and exhaustion of platelets and coagulation factors. In contrast, platelet aggregation resulting from endothelial damage plays an important role in the pathogenesis of TMA (HUS/TTP); coagulation tests such as prothrombin time, D-dimer and fibrinogen are generally normal in TMA [2]. Here, we report a case of secondary TMA that followed DIC due to severe infection resulting in acute kidney injury. Plasma exchange was performed for the treatment of TMA with favorable result in this patient.

## Case presentation

The patient was an 86-year-old Japanese woman who had been regularly visiting the Department of Neurology, Shizuoka Red Cross Hospital, for the treatment of Parkinson disease; the patient had a well-maintained activity of daily living (ADL) score. A routine physical checkup was done regularly and blood tests revealed no abnormal findings. She was brought to the hospital because of low back pain and fever, and was admitted in June 2010. On day 2 of hospitalization, she began to have difficulty in moving. The level of consciousness was E3V5M6 according to the Glasgow coma scale, and she fell into shock state; her blood pressure and pulse rate was 70/30 mmHg and 75/min, respectively, and temperature was 37.8°C; the respiration was distressed (24/min). Physical examination revealed petechial hemorrhage on the soft and hard palates, right cost-vertebral angle tenderness, and numerous ecchymoses and non-palpable petechiae of the legs. A plain abdominal radiography revealed a calculus of the right ureter.

The results of the laboratory studies on day 2 in the hospital were as follows. Urinalysis showed proteinuria and the urinary sediment contained 20–29 isomorphic red blood cells per high-power field. The amount of proteinuria could not be determined because the patient rapidly became anuric in the hospital. Leukocytosis (10,900/μL), anemia (hemoglobin level of 10.6 g/dL), and thrombocytopenia (4.5 × 10^4^/μL) were present, and immature granulocytes with cytoplasmic vacuolation and Dohle bodies were identified in the peripheral blood. Impaired coagulation was noted, including prolonged activated partial thromboplastin time (APTT, 51 seconds) and prothrombin time (PT, 18.8 seconds, 1.63 by INR), as well as increased fibrin/fibrinogen degradation products (FDP, 645 ug/mL), and plasminogen activator inhibitor 1 (PAI-1, 146.8 ng/mL). The D-dimer level, determined on day 3, was 103.4 ug/mL. Inflammatory markers were markedly elevated (CRP, 6.91 mg/dL; procalcitonin, 141.96 ng/mL). Indirect hyperbilirubinemia (1.3 mg/dL), an increase in transaminases (AST, 407 IU/L; ALT, 101 IU/L) and an elevation in the serum lactate dehydrogenase (LDH, 1160 IU/L) were also present. Laboratory data associated with renal function showed elevations in urea nitrogen (UN, 43.3 mg/dL) and serum creatinine (2.09 mg/dL). The immunological profile showed normal levels of immunoglobulin and complement, but positivity for rheumatoid factor and antinuclear antibody (ANA; ×40) was noted. Perinuclear ANCA, cytoplasmic ANCA, and anti-glomerular basement membrane antibody all tested negative. Computed tomography revealed right hydronephrosis and hydroureter due to right ureteral stone. *Proteus mirabilis* was isolated from both blood and urine cultures.

After admission, renal failure developed rapidly, and the level of her consciousness deteriorated. Intravenous piperacillin/tazobactam (PIPC/TAZ) 6.75 gram per day was started, and was subsequently switched to ampicillin (AMPC, 4 g/day) and gentamycin (60 mg/day, i.e., 1.7 mg/kg) based on the blood and urine culture results, followed further later by ciprofloxacin (CPFX, 200 mg/day). Anuria continued even despite the placement of DJ-ureteral catheter for the right ureteral obstruction. The laboratory profile on day 2 in the hospital fulfilled the diagnostic criteria of DIC [3]. A marked elevation of serum PAI-1 and a reduced protein C level were observed, indicating a secondary anti-fibrinolytic DIC state. Continuous hemodiafiltration (CHDF) was performed to remove excessive cytokines in addition to the administration of broad-spectrum antibiotics and a single infusion of antithrombin III. In response to these therapies, severe infection was successfully controlled, and DIC was well managed, as evidenced by the normalization of APTT and PT as well as the improved levels of FDP, fibrinogen and PAI-1 (Table 1). Disturbed consciousness and renal failure, however, persisted with sustained thrombocytopenia, high LDH levels, anemia, and the appearance of schistocytes.

**Table 1:** Time courses of coagulation and inflammation profile associated with microangiopathic hemolytic parameters

	**Day 1**	**Day 2**	**Day 3**	**Day 4**	**Day 5**	**Day 6**	**Day 7**
Platelet count (×10^4^/μL)	23.0	4.5	2.2	2.3	2.4	1.0	3.2
LDH (mg/dL)	217	1160	2267	1836	1412	1482	
Serum Cr (mg/dL)	0.51	2.09	3.35			3.82	4.07
APTT (seconds)		51			39	32	28
PT-INR		1.63	1.36	1.52	1.03	0.90	0.95
FDP (μg/mL)		645	259	60	30		
D-dimer (μg/mL)			103.4				
Fibrinogen (mg/dL)		206	425	537	526	566	588
Anti-thrombin III (%)		90	60	74	49		62
Protein-C (%)		<1.0		23	<1.0		32
PAI-1 (ng/mL)		146.8	148.0	172.6	24.7		38.7
Procalcitonin (ng/mL)		141.9	195.1	125.6	94.9		33.6
CRP (mg/dL)	0.66	6.91	15.79	19.77		13.53	11.73

The coagulation profiles of APTT and PT were normalized on day 6 as a result of the treatment with antibiotics and continuous hemodiafiltration (CHDF). Antithrombin III (1500 unit) was administered on day 3.

Since the patient’s infection and DIC had already been well controlled, it was conjectured that the persistent abnormalities (microangiopathic hemolytic anemia, thrombocytopenia and clinical symptoms such as neurological disturbance and renal failure) were attributable to TMA. Hence, we started plasma exchange therapy on day 7, replacing the CHDF treatment. After the commencement of plasma exchange, the level of her consciousness improved dramatically in association with several laboratory parameters including the LDH level, the platelet count and the serum haptoglobin level (Table 2). The ADAMTS-13 activities measured by the well-established assay method [4] on days 8 and 19 were 44% and 40.6% (normal, 70-120%) respectively (Table 2); the ADAMTS-13 activity measured after the improvement of all the TMA symptoms except for renal injury was also subnormal (35.5% on day 86 and 33.0% on day 220, Table 2). It is to be noted that ADAMTS-13 activity was never below 10% in our patient even during the period of high disease activity, indicating that this was not a case of typical primary TTP.

**Table 2:** Time courses of the ADAMTS-13 activity and the microangiopathic hemolytic parameters

	**Day 8**	**Day 19**	**Day 25**	**Day 31**	**Day 58**	**Day 86**	**Day 220**
Platelet count (×10^4^/μL)	1.0	11.3	12.2	23.4	7.4	7.8	4.3
LDH (mg/dL)	1069	248	247	234	294	177	108
Schistocytes (%)	6.1		3.8		0.5		0.9
Haptoglobin (mg/dL)	<10			76	42		26
ADAMTS-13 activity (%)	44	40.6				35.5	33.0
ADAMTS-13 inhibitor	(-)	(-)				(-)	(-)

**Figure 1:**
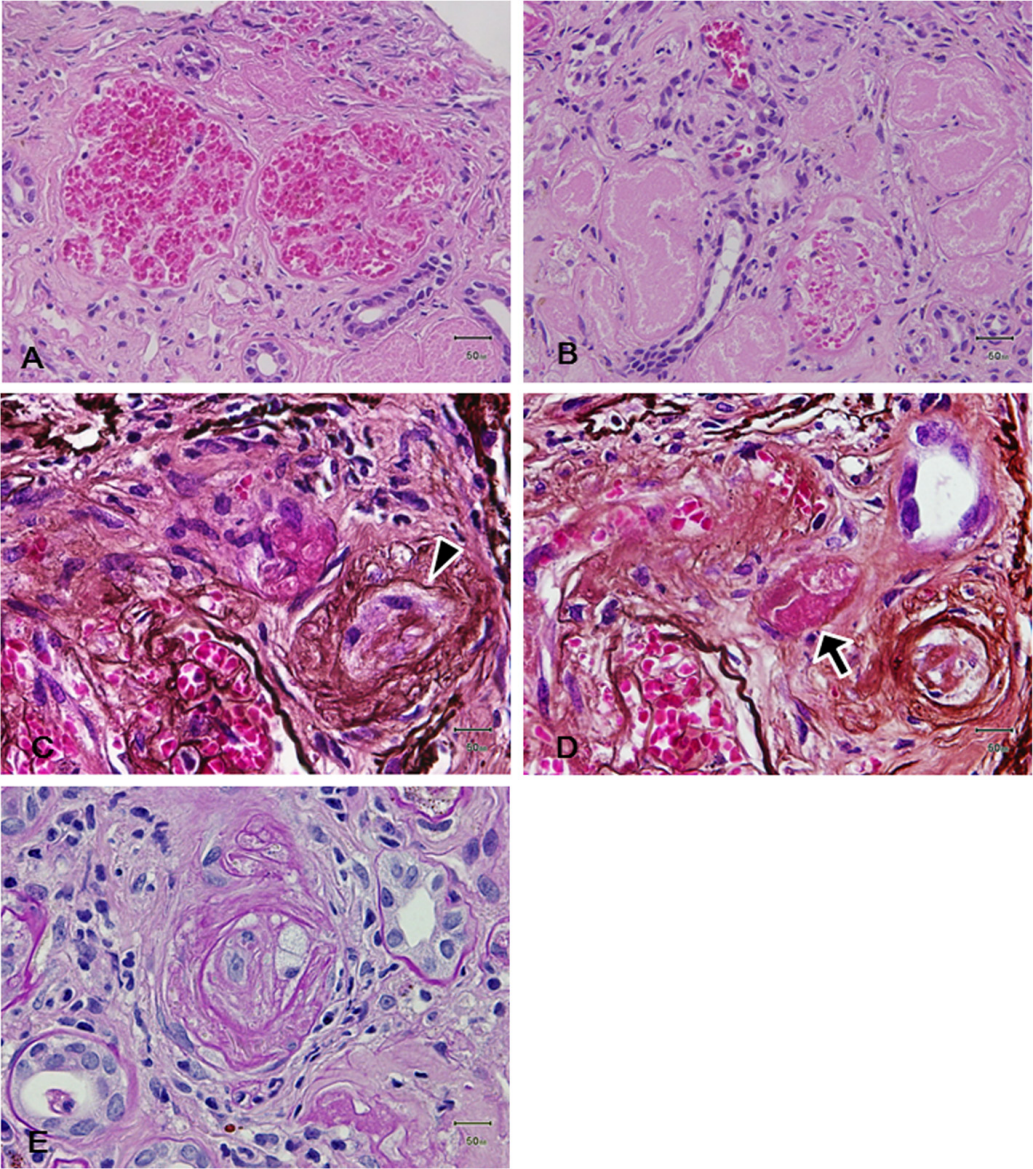
**Histopathological findings of renal biopsy. (A) **Light photomicrograph showing two glomeruli with widened capillary lumina containing red blood cells (Hematoxylin and eosin stain; original magnification, ×200). **(B) **Severe tubular necrosis with a loss of cellular detail (Hematoxylin and eosin stain; original magnification, ×400). **(C, D)** Serial sections of an afferent arteriole with obliterative intimal change (**C**; arrowhead) and intraluminal thrombus formation (**D**; arrow) (Periodic acid silver methenamin stain; original magnification, ×400). **(E)** Smaller interlobular arteries and arterioles showed occlusion or extensive narrowing of their lumen which was interpreted to represent the sequel of TMA (**D**; Periodic acid-Schiff stain; original magnification, ×400).

To determine the etiology of the renal disease which was unresponsive to plasma exchange, renal biopsy was performed on day 26. The specimen for light microscopy contained 15 glomeruli; 7 exhibited global sclerosis, 2 severe segmental sclerosis, and 2 exhibited only minor abnormalities. The remaining 4 glomeruli had dilated capillary loops that were filled with red blood cells (Figure 1-A). Aggregations of severe tubular necrosis were seen in several foci (Figure 1-B). One of these glomeruli had an afferent arteriole with occlusive intimal changes (Figure 1-C; arrowhead) and what appeared to be a small intraluminal thrombus (Figure 1-D; arrow). Arteriosclerotic fibrous thickening of the intima was seen in the larger arteries. Smaller interlobular arteries and arterioles, on the other hand, showed occlusion or extensive narrowing of their lumen which was interpreted to represent the sequel of TMA (Figure 1-E). Fibrinoid necrosis of the vessel wall was not observed. No obvious thrombi were observed in the vascular lumina except for the above-mentioned modest lesion in an afferent arteriole. Tubular atrophy and interstitial fibrosis were seen in approximately 50% of the cortical area. Immunofluorescence studies showed no immunoglobulin, complement or fibrinogen deposition in the glomeruli or the blood vessels.

**Figure 2:**
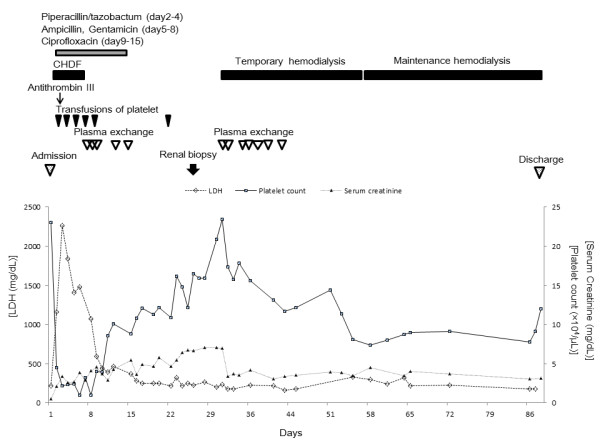
**Clinical course of the patient.** The clinical course indicated that the patient’s renal failure was irreversible, although laboratory abnormalities related to TMA improved with plasma exchange. Thus, we discontinued plasma exchange and started the patient on three-times-a-week maintenance hemodialysis beginning on day 57.

Although laboratory abnormalities related to secondary TMA improved dramatically with plasma exchange, the clinical course indicated an irreversible course of the patient’s renal failure. Hence, we discontinued plasma exchange and started the patient on three-times-a-week maintenance hemodialysis beginning on day 57. TMA did not recur after the discontinuation of plasma exchange, although moderately decreased levels of plasma ADAMTS-13 activity persisted (Table 2). On day 96, she was transferred to another hospital for rehabilitation and maintenance hemodialysis (Figure 2).

## Discussion

The present case manifested with severe sepsis caused by obstructive urinary tract infection (causative organism, *Proteus mirabilis*), and the laboratory findings showed features characteristic of DIC on day 2 of hospitalization. Despite the normalization of the coagulation abnormalities by the treatment for infection and DIC, the central nervous system symptoms and renal injury did not improve and the microangiopathic hemolytic anemia and thrombocytopenia persisted. We thus adopted the plasma exchange therapy based on the assumption that her clinical symptoms reflected the features of secondary forms of TMA rather than DIC at this point. The rapid deterioration of consciousness that took place following admission to the hospital and in the context of old age and severe sepsis could well have been a form of nonspecific delirium. However, the fact that the patient’s mental state improved dramatically with plasma exchange therapy suggests that the deterioration was part of the TMA syndrome. Consequently, significant improvements in the central nervous system symptoms and the laboratory findings were observed, although the renal dysfunction persisted. A renal biopsy performed on day 26 showed multilayering of the small arteries and arterioles with occlusive thickening of the intima. Although obvious thrombus formation was not demonstrated, except for a minute lesion in one afferent arteriole shown in Figure 1-D, these changes were interpreted as being compatible with the sequelae of renal TMA. The absence of obvious fresh thrombi could be due to the point of time of the biopsy as well as the therapeutic effects. The presence of focal severe tubular necrosis was suggestive of cortical necrosis. Although cortical necrosis often results from DIC, its presence in TMA has also been well documented, especially in HUS [5]. Although the different forms of TMA entities (primary vs. secondary) and TMA vs. DIC are difficult to differentiate histologically, the above renal biopsy findings interpreted in the context of the clinical course suggested that renal TMA superimposed on DIC led to the severe renal failure in our patient. Thus, TMA that followed DIC resulting from the severe infection was thought to contribute importantly to the disease process in the present case.

Acquired TMAs (HUS/TTP) include idiopathic and secondary cases. Secondary TMA may occur in a variety of disorders including collagen disease (e.g., SLE, rheumatoid arthritis, and scleroderma), malignant hypertension, infection caused by Shiga-like toxin-producing *Escherichia coli* (STEC) or non-STEC. The disorder is also caused by drugs such as cyclosporine A, mitomycin C, cisplatin and transplantation [6]. The autoantibody to ADAMTS-13, a specific cleavage enzyme of von Willebrand factor (VWF), was found to be involved in the development of TTP, and the congenital ADAMTS-13 deficiency due to gene mutations was reported [7]. Furthermore, under low ADAMTS-13 activity, ultra-large VWF (UL-VWF) multimers are increased because the low level of ADAMTS-13 fails to cleave UL-VWF multimers, resulting in excessive platelet aggregation and the subsequent microvascular occlusion and organ dysfunction. In the present case, physical examination and laboratory findings clearly eliminated the diagnosis of collagen vascular diseases or STEC infection. The possibility of malignant hypertension was ruled out by the pre- and post-hospitalization blood pressure records. Alternatively, systemic inflammation has been shown to be associated with ADAMTS-13 deficiency and a subsequent elevation in UL-VWF [8]. It is, thus, reasonably inferred that the TMA in the present case is attributed to severe infection.

It has frequently been reported that infectious diseases cause TMA. Douglas et al. [9] showed that infection other than STEC infection preceded TMA in 36 out of 65 cases. Coppo et al. [10] also reported the presence of infectious diseases other than verotoxin-producing *Escherichia coli* (VTEC) in 9 of 30 TMA cases. Furthermore, Booth et al. [11] reported that infectious disease other than STEC infection was identified in 31 (7%) of 415 suspected TMA cases that were registered in the Oklahoma TTP-HUS Registry treated with plasma exchange. The same authors analyzed 98 cases of TMA with infection and reported that the causative organisms included 41 species of bacteria, as well as various viruses and fungi.

Of note, among the 31 cases with TMA in the Oklahoma TTP-HUS Registry, 10 patients manifested abnormal coagulation profiles, although it remained uncertain whether these were DIC cases incorrectly diagnosed as TMA or the co-morbid state of DIC and TMA [11]. Wang et al. [12] also reported two patients with severe infection who most likely suffered from both DIC and TTP; both patients presented with renal injury, purpura, thrombocytopenia, and coagulation/fibrinolysis abnormalities characteristic of DIC, as well as a markedly reduced ADAMTS-13 activity level and positive ADAMTS-13 inhibitor. Ono et al. [13] also reported decreased ADAMTS-13 activity levels in patients diagnosed as having sepsis-induced DIC with severe renal injury, and these authors suggested that the decreased ADAMTS-13 level was attributable to the reduced hepatic production and enhanced degradation by various proteases induced by DIC. In the present case, we assume that the initial presenting clinical picture was that of DIC, and TMA was superimposed somewhat later in association with a moderate decrease (44%) in the ADAMTS-13 activity.

Interestingly, the ADAMTS-13 activity level still remained low (35.5%) at the time when the patient was discharged from the hospital when her infection was clinically in complete cure. Furthermore, TMA did not recur when her ADAMTS-13 activity was 33.0% (day 220). These observations lead us to surmise that the endothelial injury caused by severe sepsis superimposed on preexisting subnormal levels of ADAMTS-13 activity contributed to the development of TMA. Some clinical and experimental reports lend support to such surmise; although familial TTP often manifests during infancy, about one-half of cases remains undiagnosed until mid-childhood or later, with rare ADAMTS13-deficient individuals remaining free-of-disease throughout the third decade of life [14]. In an animal study using ADAMTS-13-deficient mice, Motto et al. [15] reported that TTP did not develop under the naïve condition, but the injection of Shiga toxin actually induced a condition resembling human TTP. These observations lend support to the premise that the loss of ADAMTS-13 activity may be necessary, but not sufficient for the induction of a clinical episode of TTP.

It is widely accepted that plasma exchange is an effective modality of the treatment of congenital or idiopathic form of TTP [1]. In the present case, plasma exchange was started with an assumption that TMA was superimposed on DIC based on the observation that consciousness disturbance and renal dysfunction as well as microangiopathic hemolytic anemia and thrombocytopenia persisted after the successful correction of coagulation parameters by the treatment of septic DIC. Following plasma exchange, the patient’s consciousness improved remarkably in association with partial recovery of the hematological abnormalities. Of note, Nguyen et al. [16] also reported beneficial effects of plasma exchange in sepsis associated with a decrease in ADAMTS-13 activity. Our experience suggests that TMA, which is effectively treatable with plasma exchange, should be taken into consideration in patients with septic DIC with a serious clinical course.

## Conclusions

The present case manifested with clinical and pathological features suggestive of TMA following recovery from DIC due to severe sepsis. Although much remains to be elucidated as regard to the mechanism for the development of TMA, the decreased level of ADAMTS-13 would act in concert with other detrimental factors associated with infection and DIC to induce TMA. To the extent that plasma exchange is expected to provide beneficial effects on TMA, this case should remind us of the possibility of TMA that follows sepsis-induced DIC.

## Consent

Written informed consent was obtained from the patient’s son for publication of this case report and any accompanying images. A copy of the written consent is available for review by the Editor of this journal.

## Abbreviations

TMA: Thrombotic microangiopathy; DIC: Disseminated intravascular coagulation; TTP: Thrombotic thrombocytopenic purpura; CHDF: Continuous hemodiafiltration; STEC: Shiga-like toxin-producing *Escherichia coli*; VTEC: Verotoxin-producing *Escherichia coli*; UL-VWF: Ultra-large Von Willebrand factor.

## Competing interest

The authors declare that they have no competing interests.

## Authors’ contributions

YS, EK were the treating physicians of the patient at Shizuoka Red Cross Hospital. YS has collected the clinical data and performed renal biopsy, and involved in writing the manuscript. EK is a director of Internal medicine at Shizuoka Red Cross Hospital. KK is a nephrologist at Keio University Hospital and performed the evaluation of the renal biopsy. KK made the pathological diagnosis together with AH and supervised the manuscript. AH is a pathologist and an assistant professor of pathology at Keio University Hospital. KH is a nephrologist and an associate professor of nephrology at Keio University Hospital, and supervised the manuscript. MH is a nephrologist and a professor of center for apheresis and dialysis at Keio University Hospital, and supervised the manuscript. TS is an emeritus professor of endocrinology, metabolism and nephrology at Keio University Hospital and supervised the manuscript. HI is a professor of endocrinology, metabolism and nephrology at Keio University Hospital, and supervised the manuscript. All of the authors have contributed to the preparation of the manuscript. All authors read and approved the final manuscript.

## Pre-publication history

The pre-publication history for this paper can be accessed here:

http://www.biomedcentral.com/1471-2369/14/260/prepub
